# Empowering Wellness: A Comprehensive Narrative Review of Cancer Prehabilitation from Treatment Onset to Surveillance

**DOI:** 10.21315/mjms2024.31.5.7

**Published:** 2024-10-08

**Authors:** Ahmad Mahmoud Saleh, Aishah Ibraheem Al Daragemeh, Hassanat R. Abdel-Aziz

**Affiliations:** 1 Nursing College, Prince Sattam bin Abdulaziz University, Riyadh, Saudi Arabia; 2 Department of Pharmacy, Al-Zaytoonah University of Jordan, Amman, Jordan

**Keywords:** neoplasms, preoperative exercise, continuity of patient care

## Abstract

Cancer prehabilitation, defined as a process occurring between cancer diagnosis and the onset of acute treatment, is highlighted for its ability to enhance physical and mental health results while decreasing overall healthcare costs. This summary introduces the concept of cancer prehabilitation and emphasises the crucial role of oncology nurses in rehabilitation care. The cancer treatment plan of prehabilitation requires timely and efficient assessment across the care continuum, focusing on enhancing outcomes at every stage of cancer. The battle with cancer involves three different assessments with distinct goals: i) prehabilitation evaluation before treatment, ii) early post-treatment evaluations of rehabilitation and iii) final evaluations of health promotion. Analyses and treatments for significant side effects or complications associated with the treatment specifically for prehabilitation are recommended. The roles of coordination, counseling, preparing for discharge and teaching are outlined as integral components of a cancer nurse’s responsibilities in the prevention of cancer. A literature search from March 2016 to June 2023 was conducted using the keywords ‘neoplasms,’ ‘cancer,’ ‘prehabilitation,’ ‘continuum of care,’ ‘care continuum,’ ‘patient care continuity,’ ‘epidemiology,’ ‘therapeutics,’ ‘health,’ ‘prevention and control’ and ‘guidelines as topic.’ The findings suggest that care coordinators or navigators for cancer should be educated to assess the physical and psychological status of patients once a cancer diagnosis is confirmed, particularly for those awaiting surgery at home. To enhance their competence in prehabilitation care, oncology nurses are encouraged to gain knowledge of certain tumours’ outcomes and cancer-related treatments. Additionally, improving the ability to evaluate patients’ functional status and emotional distress is crucial for oncology nurses involved in cancer prehabilitation.

## Introduction

Cancer has emerged as a significant healthcare challenge in the Middle East and North Africa, with statistics from the Economic Impact Project revealing that, in 2023, the anticipated rise in cancer incidence is substantial in Iraq, Saudi Arabia and Syria, with projections indicating an increase of over 100%. In the United Arab Emirates (UAE), the expected rise is even more significant, reaching 231%. The UAE is also predicted to experience the highest percentage increase in cancer-related mortality, with an expected 335% rise in annual deaths by 2040 compared to 2020 (1). This trend is anticipated to escalate the healthcare economic burden in the Middle East and North Africa due to the rising population of cancer survivors. In response to this expected increase, an expanding corpus of scientific data, particularly in the realm of keeping impairment at bay resulting from cancer treatments (known as cancer prehabilitation), suggests the potential for enhanced outcomes in oncology care. This approach could also contribute to reduced treatment-related morbidity and healthcare costs, thereby fostering improvements in both the psychological and physical well-being of patients (2, 3). Consequently, formulating cancer prehabilitation guidelines tailored to particular cancer types could offer a viable solution for mitigating the healthcare burden in the Middle East and North Africa.

### Cancer Prehabilitation Definition

On the cancer care continuum, a process called ‘cancer prehabilitation’ transpires between when a cancer diagnosis is made and when immediate therapy begins (4). This process involves conducting medical and psychological evaluations to identify deficiencies, establish a baseline functional level, and carry out interventions aimed at promoting both mental and physical well-being (5). The overarching objective of prehabilitation is to avoid or minimise the degree of physical limitations and emotional suffering that treatment-related side effects are expected to cause, thereby reducing the likelihood of significant disability (6, 7). Positioned as the initial phase of the rehabilitation process, prehabilitation takes place between the diagnosis and the start of immediate oncology care (8). Research suggests the potential for prehabilitation therapies to assist patients in maintaining a superior physical function status compared to individuals who did not receive prehabilitation intervention, especially prior to surgical procedures (9, 10) (Table 1). Importantly, the scope of prehabilitation interventions extends beyond the pre-treatment phase and may persist throughout survivorship (11). Consequently, the programme for prehabilitation stands as a potential benefit for patients with cancer throughout the duration of their cancer treatment.

Supportive and palliative care, on the other hand, provides ongoing physical, emotional and social support throughout the cancer journey, regardless of the treatment stage (12). It focuses on managing symptoms, improving quality of life and providing comfort. Such care can be introduced at any point during the cancer journey, from diagnosis onwards (13, 14).

### Cancer Prehabilitation Evidence

Reports indicate that prehabilitation programmes can improve outcomes for physical and mental health, leading to reduced surgical complications, treatment-related morbidity, hospital stays, re-admissions and overall healthcare costs (20–22). Among prehabilitation initiatives, cancer patients have benefited from widely acknowledged pre-surgery exercise training therapies. A comprehensive systematic review, encompassing 18 studies and involving 966 participants, was conducted to assess the efficiency of training interventions for reducing the risk of workplace injuries (23). The studies evaluated a variety of training interventions, including safety training programmes, hazard awareness training and ergonomics training. The outcomes of the review revealed that training interventions were effective in lowering the danger of workplace injuries. The pooled risk ratio for all studies was 0.80 (95% confidence interval [CI]: 0.72, 0.89), indicating that training interventions reduced the risk of workplace injuries by 20% (23). The intervention duration prior to surgery varied from 7 days to 52 days, with 21 days serving as the median. Exercise regimens implemented both under supervision and at home demonstrated improvements in patients’ physical function. The studies commonly employed aerobic, resistance or strengthening exercises, with prostate cancer programmes often including pelvic floor exercises, and lung cancer programmes focusing on breathing techniques. Depending on the type of cancer, each exercise session lasted anywhere from 15 min to 3 h.

Kwok and San Tay (24) examined the outcomes of a hospital-associated, home-based cancer prehabilitation programme for gastrointestinal and urological cancer patients planned for surgery. The programme includes medical optimisation, physical activity prescription, and measurement of compliance. Pre- and post-treatment follow-ups included outcome measures such as the 6-min walk test, 30-s sit to stand test, timed up and go test and the Hospital Anxiety and Depression Scale (HADS). The study emphasised the importance of prescribing individualised exercise plans and involving various healthcare professionals in the prehabilitation process (24).

While psychological interventions applied as prehabilitation were not found to influence factors such as hospital stay duration, problems, analgesic usage or fatality, they did impact patients’ psychological results, quality of life and somatic problems. Stress-reduction techniques frequently utilised in earlier research included unwinding methods, such as exercises, gradual relaxation of the muscles, meditation, directed imagery, analytical thinking and coping mechanisms. These methods were typically applied 1 day–2 weeks before surgery for one or two sessions, each lasting 45 min–90 min.

Despite advancements, information gaps remain, including the optimal timing, dosage and intervention response (amount of sessions and length). Evidence of the effects of prehabilitation over time is also limited. In the USA, a rehabilitation prospective monitoring model has been created and tested specifically for those with breast cancer, which focuses solely on functionality but fails to consider a psychological angle. To provide integrated cancer patient care, it is recommended to adopt multimodal approaches for prehabilitation that encompass both realms of the body and mind (25).

Contrary to the positive outcomes often associated with prehabilitation in cancer patients, as highlighted in previous review articles, a recent systematic review by Meneses-Echavez et al. (26) in 2023 found that the prehabilitation of cancer patients before treatment, specifically surgery, did not statistically alter the outcome of therapy compared to standard care. This finding suggests that further research and evaluation are needed to fully understand the impact of prehabilitation on cancer treatment outcomes.

### Tailoring Prehabilitation: A Model Tailored to Particular Cancer Types

The cancer treatment plan of prehabilitation emphasises prompt and effective assessments across the entire medical continuum to enhance outcomes at each stage of cancer. Three key assessments during the cancer journey include assessments conducted prior to therapy, throughout treatment and early after treatment to promote rehabilitation and health. These assessments aim to establish intervention goals based on cancer-related treatments and consider various aspects, such as functional, physical, mental, dietary and occupational adjustment status (27).

Preventive interventions for specific cancer types involve tailored assessments and remedies for problems associated with therapy. For example, patients with head and neck cancer should undergo swallowing and cervical range of motion assessments, while those with breast cancer should be assessed for shoulder and cervical ranges of motion, and nutritional status should be considered for patients with pancreatic cancer. Although specific interventions have been recommended, their outcomes have yet to be proven and the development of a standardised paradigm of care for particular cancers is ongoing (28).

### The Role Oncology Nurses Play in Prehabilitation Care

As primary caregivers of cancer patients on a daily basis, nurses enact a pivotal task in tumour rehabilitation, thus facilitating prompt assessments of their functional status. Their responsibilities encompass a range of activities, including evaluation, instruction, counseling, discharge preparation and coordination. In the context of prehabilitation, the proactive evaluation of a patient’s functional and psychological well-being before active treatment takes precedence over identifying impairments post-treatment.

In the function of counseling and education, oncology nurses help patients acquire workout routines and stress-reduction strategies to proactively prevent psychological difficulties. They also act as coordinators to aid patients following their transition from hospital to home care by discovering diverse needs and coordinating with professionals from various disciplines to ensure sophisticated evaluation and treatment.

To enhance the attributes of care in tumour prehabilitation, based on the study findings, the authors recommended bolstering cancer nurses’ knowledge regarding cancer-related treatments’ immediate and long-term impacts on treatments tailored to specific cancer types. Given the importance of functional status assessments and exercise in prehabilitation, in addition, the author also suggested to impart basic educational abilities that promote physical movement, such as motivational conversations, to educate cancer patients before treatment. Additionally, prior to treatment, a psychological evaluation is recommended to identify which people require assistance.

Oncology nurses typically provide education to cancer patients just a day before treatment, but the outcomes of such education have not been measured, and the timing for patients to improve their physical and psychological functions may be inadequate. To meet the goals of prehabilitation care, cancer care managers or navigators should be trained to assess patients’ physical and psychological health promptly after cancer diagnosis. This is particularly crucial for those awaiting surgery at home, enabling baseline function assessment and the implementation of a standardised education programme to alleviate stress and anxiety, and enhance physical function through exercise.

## Conclusion

Existing research indicates that prehabilitation programmes improve health outcomes for cancer patients after surgery and that tailored interventions for cancer types are crucial. Oncology nurses play an important role in developing prehabilitation care approaches for distinct cancers within clinical settings to reduce healthcare burdens, lower costs and enhance care quality. Prehabilitation therapies that include both physical and psychological aspects are advised. To improve nurses proficiency in prehabilitation care, oncology nurses should receive training about cancer-related therapies and outcomes for certain cancers and learn how to boost their capacity to assess patients’ functional state and psychological distress.

## Figures and Tables

**Table 1:** Prehabilitation interventions for cancer patients

Cancer type	Intervention	Outcomes	Reference (Year)
Rectal cancer	Prehabilitation exercise programme (aerobic and strength training)	Improved cardiorespiratory fitness, muscle strength and functional capacity; reduced fatigue	Chow et al. (2020) (15)
Breast cancer	Prehabilitation programme combining exercise, nutrition counseling and psychological support	Reduced surgery-related complications, improved physical function and quality of life	Schmitz et al. (2019) (16)
Esophageal cancer	Prehabilitation programme with inspiratory muscle training and nutritional counseling	Improved lung function, nutritional status, and tolerance to treatment	Liang et al. (2018) (17)
Head and neck cancer	Prehabilitation programme with swallowing exercises and nutritional counseling	Improved swallowing function, nutritional intake and quality of life	Amano et al. (2017) (18)
Lung cancer	Prehabilitation programme combining aerobic exercise, strength training and smoking cessation counseling	Improved exercise capacity, muscle strength and reduced risk of postoperative complications	Song et al. (2016) (19)
